# Neuropsychological screening of children of substance-abusing women attending a Special Child Welfare Clinic in Norway

**DOI:** 10.1186/1747-597X-5-17

**Published:** 2010-07-20

**Authors:** Bjørg Hjerkinn, Morten Lindbæk, Idar Skogmo, Elin Olaug Rosvold

**Affiliations:** 1 Addiction Unit/Research Unit, Sørlandet Hospital, Servicebox 416, N-4604 Kristiansand, Norway; 2 Institute of Health and Society, Faculty of Medicine, University of Oslo, P.O. Box 1130 Blindern, N-0318 Oslo, Norway; 3 Department of Psychiatry, Sørlandet Hospital, Servicebox 416, N-4604 Kristiansand, Norway

## Abstract

**Background:**

Exposure to alcohol and illicit substances during pregnancy can have an impact on the child for the rest of his/her life. A Special Child Welfare Clinic (SCWC) in Norway provides care for pregnant women with substance abuse problems. Treatment and support are provided without replacement therapy.

**Methods:**

We performed a neuropsychological screening of 40 children aged four to 11 years whose mothers had attended the SCWC during pregnancy, and of a comparison group of 80 children of women without substance abuse problems. The children were presented with tests chosen from Wechsler Intelligence Scale for Children, third version (WISC-III), Nepsy, Halstead-Reitan and Raven's Progressive Matrices, Coloured Version. The tests were grouped into five main domains; (1) learning and memory, (2) visual scanning, planning and attention, (3) executive function, (4) visuo-motor speed and dexterity and (5) general intellectual ability

**Results:**

No children in the study had test results in the clinical range in any domain. Bivariate analyses revealed that children of short-term substance-abusing mothers (who stopped substance abuse within the first trimester) had significantly lower test scores than the comparison group in three out of five domains (domain 2,3,4). Children of long-term substance abusers (who maintained moderate substance abuse throughout pregnancy) had significantly lower test results than the comparison group in one domain of the test results (domain 1). All but one child in the long-term group were or had been in foster homes. Most children in the short-term group stayed with their mothers. Multivariate regression analyses revealed that foster care minimum 50% of life time was associated with better scores on domains (1) learning and memory, (2) visual scanning, planning and attention, and (3) executive functions, while no significant associations with test scores was found for substance abuse and birth before 38 weeks of gestation.

**Conclusion:**

Children raised by former substance abusing mothers scored worse on the neuropsychological screening than children who had substance abusing mothers and mostly were raised in foster homes. This indicates that it is important to focus on the environment in cases where help and support are provided to presently or formerly addicted women raising children.

## Background

The offspring of substance-abusing mothers are exposed to various health risks [1-3]. Prenatal risk factors, such as substance abuse by the mother during pregnancy and her general health condition at the time of conception, can affect the development of the child. A well-documented risk factor is alcohol abuse, which may cause fetal alcohol syndrome (FAS) or other fetal alcohol spectrum disorders (FASD) [4].

It has been shown that both the amount of alcohol and the pattern of alcohol use are important when considering the risk of damage to the fetus [5]. The development of children who were exposed to illicit substances may also be severely impaired and the consequences of prenatal substance exposure in the first years of life are well documented [6-9]. Drug exposure in utero may produce transitory effects, which will likely not be constant across different age groups. Some negative effects may not be apparent or testable until the area of the CNS (central nervous system) underlying the behaviour in question is sufficiently developed [10]. Various postnatal risk factors resulting from the hazardous life circumstances of substance-dependent parents also influence the health outcomes in these children [11].

The results of prospective studies of the long-term effect of prenatal substance exposure are inconsistent [12,13]. Environmental factors can have important influence on the development of children prenatally exposed to substances [14-16]. Attention deficit and hyperactivity disorder (ADHD) is often mentioned during the investigation of children of substance-abusing mothers [17]. However, the diagnosis of ADHD is complicated by the frequent occurrence of co-morbid conditions, such as learning disability, conduct disorder and anxiety disorder. Children that feel unsafe in their environment may also develop symptoms that mimic ADHD, and psychosocial interventions may be the preferred treatment if co-morbid disorders are present [18].

In 1994, the health authorities of the county of Vest-Agder, in co-operation with central health authorities, decided to set up a Special Child Welfare Clinic (SCWC) in Kristiansand, Norway. It started as a project; however, three years later this endeavour became a permanent service. The intention was to provide treatment and support to substance-abusing pregnant women and their children. The aim of the treatment was to prevent adverse effects of substance abuse on the children by enabling the women to stop substance abuse as soon as possible, without replacement therapy. The intention was thus to ensure the children a safe childhood environment by supporting the mothers. Replacement therapy with methadone or buprenorphine is the current recommendation for pregnant substance abusers in many countries [19,20]. However, pregnancy in substance abusers is not an indication for opioid replacement therapy in Norway. Moreover, there is no available replacement therapy for substances such as amphetamines.

The substance-abusing women attended the clinic frequently, their special needs were mapped and an individual plan of action was prepared. Substance abusers who stopped this behaviour before the end of the first trimester (short-term users) had birth outcomes that were similar to those of mothers with no substance abuse, whereas mothers who continued their substance abuse during pregnancy (long-term users) were more likely to experience premature birth and give birth to children with a low birth weight (< 2500 g) compared with women in the comparison group [21]. It was anticipated that frequent appointments at the SCWC after the child was born would support and help the mother and provide the children with a safe environment in which to grow up. The aim of the SCWC was to evaluate the mothers' parenting abilities and to provide resources to guide these women, when necessary.

The purpose of this paper is to present the results of the neuropsychological screening of children whose mothers attended the SCWC in Kristiansand. Our aim was to assess whether there were differences in neuropsychological performance among the children of mothers who stopped substance abuse early in pregnancy (short-term users), the children of mothers who continued using substances throughout the pregnancy (long-term users), and a comparison group of children of mothers with no abuse of substances. It was anticipated that children of short-term users would score better on the tests than children of long-term users as the first group had better neonatal outcome [21]. As most women attending the clinic were poly substance abusers, the tests assessed the different types of outcomes that we thought would be affected by prenatal substance exposure, based on the literature and our own experience [22-25]. We designed the tests to capture qualities such as attention deficit, memory, visuo-motor functioning and executive functions. This study was meant to be a screening and not to represent a complete diagnosis of the children. It was also important that children of all ages complete the tests; therefore, we aimed to develop a tool that was engaging enough to motivate the children to complete the tests.

## Methods

### Participants

During its first eight years of operation, the SCWC in Kristiansand was in contact with 102 substance-abusing pregnant women. We searched the diagnosis registers from the maternity and paediatric wards at the local hospital for diagnoses of substance addiction, substance abuse, neonatal abstinence syndrome, convulsions and cramps. This search revealed that only one such pregnancy did not result in contact with the SCWC during this period.

Of the 102 women, 13 had abortions, two had children who were adopted, 27 had only a brief contact with the clinic and one did not want to participate in this investigation. The remaining 59 women attended the SCWC during pregnancy and until their children were at least two years of age or were placed in alternative care. The 62 children of these women (SCWC children) and 169 children of non-addicted mothers (comparison group children) constituted the basis of this investigation. The mothers who had attended SCWC were contacted by phone and asked if they would be in the investigation. The comparison group was chosen randomly from a child welfare clinic located within the premises of the SCWC and from two schools located in the centre of Kristiansand. The non-addiction status of the mothers in the comparison group was assessed by self report of substance use.

As the purpose of this investigation was to evaluate the neuropsychological development of the children, we only included children aged four to 11 years (i.e., born between 1994 and 2000). Forty-four children in the user group were born during this period. The mothers of two children did not want them to be tested and two children were unable to perform the test, which left 40 (91%) children in the user group. The study sample of this report therefore consists of 40 SCWC children aged four to 11 years and 80 children of the same age, who were chosen randomly from the comparison group.

The user group was divided into two groups: the "short-term use group" included women who stopped the substance abuse before the end of the first trimester and the "long-term use group" included women who reduced their substance abuse habits considerably, but continued to use substances from time to time throughout the pregnancy.

Selected background data on the 120 children who participated in the neuropsychological screening are listed in Table 1. Data on the total material are provided in previous articles [21,26].

**Table 1:** Background data on the children in SCWC

	Short term n = 22	Long term n = 18	Substance abuse total n = 40	Comparison n = 80
	n (%)	n (%)	n (%)	n (%)
Biological mothers				
Education < 9 years	19 (86)	13 (72)	32 (80)	7 (9)*
Social benefits	8 (36)	12 (67)	20 (50)	0 (0)*
Children				
Birth at < 38 weeks gestation	4 (18)	6 (33)	10 (25)	18 (23)
Gender Male	11 (50)	8 (44)	19 (48)	43 (54)
Foster home for min. of 50% of lifetime	2 (9)	13 (72)	15 (38)	0*
	Mean (SD)	Mean (SD)	Mean (SD)	Mean (SD)

Maternal age in years	25 (5.2)	25 (5.6)	25 (5.3)	31 (5.1)
Age in years of child when tested	8.1(1.9)	7.7 (1.9)	7.9 (1.9)	8.1 (1.8)

*Substance Substance abuse group/control (Cross table analyses, Pearson chi-squared value) *P *< 0.001

### Design

The neuropsychological screening was part of a larger study investigating pregnancy and birth outcome of mothers who had attended SCWC. The SCWC women were contacted by phone and asked if they would be in the investigation. The comparison group was recruited by asking parents from a child welfare clinic on the same premises if they would agree to be in this investigation. For children in school information about the investigation was sent home with the children and the parents were later asked if they would agree to participate.

The SCWC children were exposed to polysubstance abuse early in pregnancy. The specific amount of substances used by the mothers was not registered; however, various substance abuse services were well acquainted with all of these women and knew that they all had serious substance abuse problems. In the user group, 22 women stopped abusing substances before the end of the first trimester (short-term users). The remaining 18 women reduced their abuse considerably, but continued to use substances from time to time throughout the pregnancy (long-term users). We segregated the substance abuse group in this way because we had found differences between the two groups regarding several variables that were registered during pregnancy and child birth [21]. Comparisons were performed using children, aged between four and 11 years, randomly selected from the total comparison group.

Data were collected from the medical records of the mothers at the SCWC and using a self-administered questionnaire that was filled in by the mothers or by foster parents. The neuropsychological screening of the children was performed by a trained psychologist. In addition to data from pregnancy check-ups, the medical records contained the socio-economic data of the mothers at the time of pregnancy and childbirth.

The substance abuse of the mothers was registered via self-report while attending the SCWC and was confirmed by members of interdisciplinary groups and by urine specimens submitted during pregnancy. In a few cases, there was disagreement between self-report in the questionnaire and the medical records, which often included urine analyses. The information from the medical records was considered more reliable and was used in our analyses. The questionnaire contained questions regarding the pregnancy, birth and birth outcome, socio-economic issues at the time of screening and substance use during pregnancy. Births before week 38 was registered as substance abusers are known to give birth 2 to 3 weeks prematurely [27]. Children born prematurely may have poor cognitive development.

Some of the mothers in the comparison group had experimented with substance use. However, none of them had problematic use.

Information pertaining to the level of education of the biological mothers and reception of social benefits at the time of pregnancy was obtained from the medical records. In the comparison group, these recordings were performed at the time of the screening. It was anticipated that the women in the comparison group had all finished junior high school before their children were born. The level of education was dichotomized into "≤ 9 years" (junior high school) or into "started high school or higher education". The social benefits variable was dichotomized into "yes" or "no"; the "no group" included being employed full time or part time, studying or being in work training. Time spent in foster care was registered at the time of screening. Some children had been in foster care temporarily. To investigate the influence of foster care over time, we dichotomized the variable into having spent > 50% of the life in foster care or not.

The study protocol was assessed by the Regional Committee for Medical Research Ethics and was approved by the Norwegian Data Inspectorate. This study was conducted in full accordance with the World Medical Association Declaration of Helsinki.

### Setting

The testing was performed in 2005 and 2006. The parents of all SCWC and comparison group children provided written consent to participate in the study. The parents of children under school age were contacted by phone and appointments for the screening were made. A note containing information on the time of testing was sent to the parents of school children; we also asked these parents whether they wanted to be present during the screening of their child. We then went to the school on random days and performed the tests on pupils chosen at random among those who had agreed to participate. We stopped recruiting when the size of the comparison group was twice that of the user group. This ratio has been shown to strengthen the analysis [28,29] and was within the limits of this investigation. The age of the comparison group children was matched to the SCWC children's ages.

Children of all groups were tested in the same rooms. Children under school age were tested at the child welfare clinic; the older children were in the schools. The tests were administered using the same sequence for all subjects. Children in the user groups obtained appointments on the same days as the children in the comparison group. The testing psychologist was blinded to the nature of the groups being tested. All participants were tested by the same psychologist, who was well experienced in the testing of children. After completion of the tests, the children were given a small but popular gift that was chosen from a bag.

### Measures

The various neuropsychological tests were selected from the WISC-III [30], NEPSY [31] and Halstead-Reitan [32]. Raven's Progressive Matrices, Coloured Version [33] were also used.

We aimed to use tests that were validated for children aged four to 11 years. The raw scores were transformed into standardized scores using the mean and standard deviation (SD) of the comparison group in the age ranges of four to five years, six to seven years, eight to nine years and 10 to 11 years. For the purpose of the analyses, the standardized measures of the tests were grouped into five different cognitive domains, as is often done in neuropsychological investigations of children [34-36], and summary scores were compiled. The domains were (1) learning and memory, capturing working memory and learning; (2) visual scanning, planning and attention, capturing the speed at which visual stimuli are translated and recognized as something that makes sense; (3) executive functions, capturing superior integration systems, ability to adjust and mental flexibility; (4) visuo-motor speed and dexterity, capturing the ability to synchronize visual and motor impressions; and (5) general intellectual ability, as shown in Additional file 1.

The TMT A (trial making test) and TMT B and Visual attention tasks were too difficult for children aged four to six years. The domain visuo-motor speed and dexterity were calculated for children older than six years. For the younger children, we computed sum scores for the Grooved pegboard. For the other domains, scores were obtained from the results available on each child.

The researchers made efforts to ensure that children with a test profile in the borderline range were in contact with other initiatives.

### Statistical analyses

Data were analysed with bivariate techniques (*t *test and chi-squared test) (Table 1), ANOVA (Table 2) and general linear regression (GLM) (Table 3) using the SPSS version 16 program. Significance was set at *p *≤ 0.05.

**Table 2:** ANOVA analysis of the z-scores of the substance abusing groups and comparison group by cognitive domain

	Short term		Long term		Comparison		Short-term/ comparison	Long-term/ comparison
	Mean (n)	SD	Mean (n)	SD	Mean (n)	SD	*P *value	*P *value
Visuo-motor speed and dexterity	-0.057 (16)	0.627	-0.328 (16)	1.359	0.0 (57)	0.697	0.915	0.358
Visual scanning, planning and attention	-0.543 (20)	0.462	-0.172 (17)	1.103	0.0 (80)	0.690	0.010	0.655
Learning and memory	-0.702 (22)	0.880	-0.476 (18)	1.012	0.0 (80)	0.682	0.001	0.060
Executive functions	-0.810 (22)	0.829	-0.485 (17)	0.799	0.0 (80)	0.708	< 0.001	0.046
General intellectual ability	-0.544 (22)	1.352	-0.609 (17)	1.444	0.0 (79)	0.986	0.115	0.111

**Table 3:** Linear regression analysis of some cognitive domains and registered factors known to have influence on the development

Environmental factor			Learning and memory			Visual scanning, planning and attention			Executive functions		
		n	B	95% CI	p	B	95% CI	p	B	95% CI	p
Substance abuse	Yes	40	-.264	-.687 to .158	.218	.090	-.319 to .499	.665	-.396	-.817 to .024	.064
	No	80	0			0			0		
Foster care min. 50% of lifetime	Yes	15	.506	.016 to .997	.043	.725	.246 to 1.203	.003	.488	0 to .976	.050
	No	105	0			0			0		
Born before week 38	Yes	28	-.364	-.689 to -.040	.028	-.260	-.566 to .046	.095	-.226	-.549 to .096	.167
	No	92	0			0			0		

## Results

No children had test results in the clinical range, which is considered as > -2 SD, in any domain. In all three groups, some children had test scores in single tests that were in the clinical range.

The average testing time was 60 min (SD, 12 min) in the long-term use group, 57 min (SD, 10 min) in the short-term use group, and 56 min (SD, 6 min) in the comparison group.

No significant differences in testing time were found among groups as the ANOVA analysis of differences showed mean difference_long-term/comparison _-0.266, p = 0.700, mean difference_short-term/comparison _-0.176, p = 0.825. None of the groups exhibited differences in eventual tendency for vigilance, which is the decline in performance that may occur after a sustained cognitive demand and may, therefore, influence the test results. For instance, we found no differences in the results of tests presented in the beginning, in the middle or at the end of the sessions.

An ANOVA analysis (Table 2) showed that children of mothers in the short-term group had significantly lower test results (i.e., they tested more poorly) than the comparison group in three domains: (1) learning and memory, (2) visual scanning, planning and attention and 3) executive functions. The long-term group only had significantly lower results than the comparison group in (3) executive functions. No differences were found between the user groups and the comparison group in (4) visuo-motor speed and dexterity and (5) general intellectual ability.

**Figure 1:**
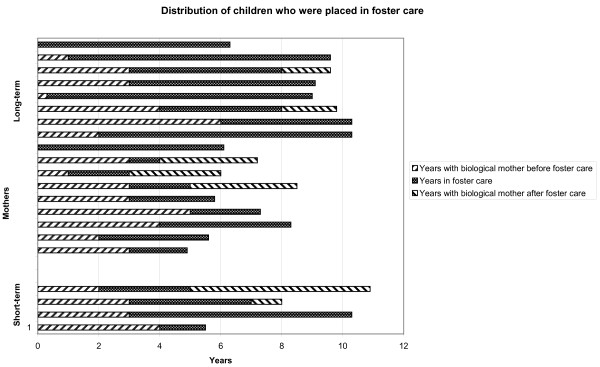
**Overview of placement in foster care**. Distribution of children of mothers in the short-term user group (bottom) and long-term user group (top) who were placed in foster care. Age (years) when placed in foster care, years in foster care and years with biological mother for the children who were no longer in foster care.

At the time of investigation, 17 (94%) children in the long-term use group and four (18%) children in the short-term group had been in foster care. Fifteen (38%) children had been in foster care for at least 50% of their lives (Fig. 1). Four children in the long-term group and two children in the short-term group had been in foster care for more than a year, but had been returned to their mother. One child in the long-term group had been in foster care for less than a year. Three children who were placed by child welfare services with their biological father (who had no history of substance abuse) are included in this percentage. No children in the comparison group were or had been in a foster home. The average age of placement in a foster home was 2.6 years (SD 1.7 years) in the long-term use group and 3.0 years (SD 0.8 years) in the short-term use group. All the mothers who lost custody of their children did so because of relapse to heavy substance abuse. More mothers in the long-term use group had support from social benefits when the child was born compared with the short-term group, and twelve (80%) of them had their children in foster care at the time of testing. Among the children of the 20 mothers who received social benefits at child-birth, 12 (67%) children had been in foster care for a minimum of 50% of their lives, whereas this was true for 13 (72%) children in the long-term group and for two (9%) children in the short-term group.

There was a high correlation between long-term substance use and placement in foster care (Spearman's rho, 0.986, *p *< 0.001). We analysed the test results using foster care for at least 50% of their lives as the factor variable in ANOVA. The z-scores observed for the children in foster care were not significantly different from those of the comparison group, in any domain, whereas SCWC children living with their biological mother had significantly lower scores compared with the comparison group, in the following domains: (1) learning and memory (difference, -0.772; SD, 0.910; *p *< 0.001); (2) visual scanning, planning and attention (difference, -0.633; SD, 0.577; *p *< 0.001); (3) executive functions (difference, -0.819; SD, 0.834; *p *< 0.001); and (5) general intellectual ability (difference, -0.636; SD, 1.41; *p *< 0.040). No significant difference was found in domain (4) visuo-motor speed and dexterity (difference, -0.245; SD, 1.16; *p *= 0.404).

General linear regression analyses with the cognitive domains in which there were significant differences in the ANOVA as the dependent variables, revealed that the substance use group was not significantly associated with the test results in any domain, while being in foster care was associated with significant better screening results in all three domains (Table 3). Being born before 38 weeks of gestation were significantly associated with low test results in the learning and memory domain.

At the time of the testing, four mothers had received replacement therapy with methadone or buprenorphine; one (5%) of them was in the short-term group and three (17%) of them were in the long-term group. Pearson chi-square 1.61, *p *= 0.204.

## Discussion

The purpose of this study was to investigate the neuropsychological outcome of children ages four to 11 years old whose mothers had attended the SCWC during pregnancy and the child's first two years of life. The screening was designed to capture neuropsychological functions connected with prenatal substance exposure, such as attention deficit and learning problems. All children had test results in the normal range in all domains. Some children in both the user and the comparison groups had test results in the clinical range (> - 2 SD), but only in single tests. Children of substance-abusing mothers often have impaired development [6]. The results of our study with all children scoring within the normal range of the domains, suggest that the children benefited from their mother's attendance at the SCWC, where emphasis is placed on stopping or reducing substance use. However, the age range of the children was wide and they were still quite young (mean age at testing, 7.9 years); adverse effects of substance abuse may manifest at an older age.

We wanted to study if there were differences in the test results between the user groups and the comparison group. The ANOVA analysis showed significantly lower test scores in the short-term use group than the comparison group regarding the domains (1) learning and memory, (2) visual scanning, planning and attention and (3) executive functions. This may point to some disturbed function in children of substance abusers. Test scores in the lower range of many different domains indicate impaired development. These three domains are frequently correlated with attention deficit and hyperactivity [37,38] and with learning difficulties [39-41].

The linear regression analyses revealed, however, that being in foster care for more than 50% of lifetime was the only factor that was significantly associated with the screening results in the three domains (1) learning and memory, (2) visual scanning, planning and attention and (3) executive functions. Children of mothers in the short-term use group mostly lived with their biological mothers and they were probably exposed to a significant amount of negative environmental factors that may have disturbed their development. An unsafe environment may stem from the mother having a limited network, i.e., few friends who do not use substances, and from the mother often changing addresses and partners. Lack of cognitive stimulation may also contribute to a less satisfactory performance in the neuropsychological screening.

There was no difference among any of the groups regarding the time necessary to complete the test or vigilance. This was initially not expected because of the relatively short time used to perform the test tasks. This result indicates that all children participated well and endured the situation surrounding the testing in a satisfactory manner.

Children of long-term users did not differ significantly from the comparison group on the test results in the bivariate analyses, with the exception of "executive functions". This was in contrast with the neonatal findings, which demonstrated that long-term users were more likely to experience adverse birth outcomes compared with the comparison group [21]. Fetus in the long-term use group had been exposed to various doses of substances at irregular intervals throughout the pregnancy. These children were more often premature and a significantly larger proportion of them had a birth weight < 2500 g. There are few reports on the development and environmental conditions of children that are prenatally exposed to illicit substances, either early in pregnancy or from time to time throughout pregnancy [42]; however, different neuropsychological problems have been observed, such as decreased general cognitive functioning and deficits in learning and memory tasks [6]. One important issue in the recommendation of opioid replacement therapy in pregnancy is the prevention of variations in the concentration level of substances as such variations may cause adverse reactions in the fetus. In our study, the fetus in the long-term use group had been exposed to various doses of substances at irregular intervals throughout pregnancy. However, the substance abuse during pregnancy was monitored and found to be considerably reduced, even in the long-term group, and only one newborn needed treatment for neonatal abstinence syndrome [26].

The environment in which children are raised seems to be one of the most important factors of determination of their development [11]. Foster homes are thought to ensure the safety of children and to help optimize their development. In Norway in 2006 2.6% of children 0-17 years received help from the Child Welfare System, 0.4% were placed in foster care. Of the SCWC children, 21 (51%) were or had been in foster care. This is an indication that the children are born into an environment of great risk.

The results of the neuropsychological screening indicate that adverse environmental factors had a negative association with the development of SCWC children that was as significant as the prenatal exposure to substances.

In early stages, and primarily before the age of five, the plasticity of the child's brain allows responding to a good and safe environment [43]. The average age of the children placed in foster care in our study was 2.6 years; thus, they had the possibility to compensate for prenatal adverse effects of substance abuse.

The multivariate analyses revealed that being in foster care were associated with positive test scores in all the three domains where we found significant differences in the bivariate analyses. Being born before 38 weeks of gestation was associated with low test scores in the domain learning and memory. This might be related to the adverse birth outcome of the long-term substance abusing group as mothers who used some substances throughout pregnancy more often gave birth before 38 weeks of gestation and more often relapsed to substance abuse after the child was born [21].

We have not measured environmental factors in this study. However the results indicate that other elements than the use of substances in pregnancy influence the development of the children. Other confounding factors may not have been discovered in this sample.

One important aim of the SCWC is to try to optimize the development of the children by ensuring them a safe environment in which to grow up. The close monitoring of parents and infants in the first years of the child's life may have contributed to the discovery of adverse conditions and, thus, was instrumental in placing the child in foster care. The long-term use mothers were often asked by the child welfare service to continue to provide urine specimens after the child was born, which allowed the easy detection of relapse to substance abuse. The support and guidance of the SCWC, in addition to the help provided by other initiatives, may have contributed to the enhancement of the mothers' parenting skills during the first years. As the child grows older, the demands on the mother's parenting skills change. The child will challenge the mother in a way that calls for determination and attention. This might cause stress and relapse to heavy substance abuse, leading to the need for foster care for the child.

Also among the short-term users, contact with the SCWC may have helped identify children living in high risk environments and mothers' relapse into substance abuse. In cases where adverse conditions were suspected, it was, however, often difficult to make the mother realize that it would be good for the child's development if she agreed to receive help and guidance. In cases where substance abuse or major neglect was not present, any help was dependent on the mothers' consent. The test results indicate that children in the short-term use group had experiences in their environment that may have had negative influence on their neuropsychological development. Our study shows that a stronger effort is needed to follow children who are being raised by former substance-abusing mothers.

Overall, four mothers had received replacement therapy at the time of the children's testing. All had temporarily lost custody of their children because of relapse to substance abuse and got their children back after being stabilized. Fear of losing their children may be a reason why the craving for substances is often a problem that the users try to hide from the SCWC personnel. Therefore, replacement therapy rarely becomes an issue. A more open discussion of this topic may have been helpful to try to avoid placement in foster care.

In Norway, most of the resources necessary to help mothers with special needs are available through the child welfare system. This investigation indicates that it is of great importance to pay extra attention to a child of a substance abuser, even if the mother has managed to stop her substance abuse behaviour. It is crucial to realize that it is necessary to provide help and support to the mother to prevent problems for the children as they grow older.

There were some limitations to this investigation. There was no randomization and no fixed treatment program. Randomization was not possible, as each user who got in contact with the SCWC had to be helped, for ethical reasons. Pregnant women were given individual treatment, according to their needs. Help was also provided by other institutions, such as the social welfare system, the child welfare service, substance abuse counselling and community health services or the hospital. However, SCWC had the main responsibility of following up the medical issues related to the pregnancy, and the clinic had the resources necessary to give the mothers more attention than is given at an ordinary child welfare clinic.

Another limitation of this study was that we did not register the exact amount of substances used during pregnancy. The women who attended the SCWC were, however, known to have serious substance abuse problems, according to various social and substance abuse services in the community. Urine specimens were used to confirm the cessation of substance use.

Some environmental factors that might be important confounders are not in the analysis. All, but one child in the user groups lived with their mother in single parent families where the mother often had had several new partners, while all children in foster homes lived in two parents families. In the comparison group 20% of the children lived in single parent families.

The home environment has not been measured in the study. If adverse environmental conditions were discovered by the SCWC, necessary resources, such as home based intervention, help with respite care or economic support to allow the child to be placed in a nursery school, were provided by the Child Welfare System, if the mother agreed. Before a child is placed in foster home, voluntary measures should be tried as long as there is no direct danger to the child. Most mothers in the short-term use group did, however, not accept SCWC' offers of support.

The home environment in the foster homes was not measured, but we consider that they offer a good environment for the child.

The age range of the children was broad. To recruit a number of children that was sufficient for statistical analysis, we investigated the first eight years of operation of the SCWC. We made an effort to use tests that were validated for the specific age range. Moreover, z-scores were calculated for different age groups to be able to compare the results across ages. In the analyses, the age of the children did not influence the results.

The sample size was small. We wanted to investigate children older than four years of age, as the establishment of special child welfare clinics is now recommended in all municipalities of Norway and little is known about the development of the children who attend these clinics. A larger sample size was not within the scope of this investigation. The significant differences observed between the groups in the test scores may be considered as being reliable. However, important differences may have been overlooked. It is important to consider our results in the light of the settings of the SCWC. Additional research on the children of mothers with substance abuse living in Scandinavia is required, as the treatment is often given in small local initiatives in these countries.

The strength of this study was that we succeeded in establishing contact with all but one pregnant substance-abusing woman within the relevant region, and that all but two of the children in the target age group participated in the neuropsychological screening. Although the identities of the children of substance-abusing mothers were known to the main investigator, they were not revealed to the psychologist who performed the neuropsychological screening. We intended to ensure that the result of the screening was not influenced by the identity of the subjects. However, a fully blinded investigation was not within the scope of this research.

We anticipate that initiatives such as the SCWC will be important to support substance abusing women in their efforts to stay free of substances and to help and advise them on how to parent their children. Additional follow-up investigations are needed to assess how to prevent impaired neuropsychological function in children born and raised by substance-abusing mothers. In order to help mothers with substance abuse problems to become better parents a screening of early bonding and attachment could be of use. Guiding, for instance by means of Video-feedback Intervention (for instance Marte Meo [44]) in order to encourage the mothers to use "their own strength" to advance and stimulate developmental processes. If the mother/child attachment is strong it will be easier for the mothers to make good decisions regarding the child's home environment [45].

## Conclusions

The children of mothers attending the SCWC scored within the normal range of the neuropsychological tests administered between the ages of four and 11. Children living with their biological mothers, who mostly had stopped their substance abuse before the end of first trimester, had significantly lower test scores compared with the comparison group in three domains related to attention deficit problems and learning problems. The test scores were better for children living in foster homes, even if most of these children had mothers who continued their substance abuse throughout the pregnancy and also had more premature births than the comparison group. This finding suggests that the development of the children may be more related to the environment in which they grow up than to the exposure to moderate amounts of substances during pregnancy.

Although the early cessation of illegal substances had a positive influence on birth outcomes the follow up of mothers and children in the short time users group seems not to have been sufficient. The health authorities of Norway recommend the implementation of special child welfare services in all municipalities, to prevent the adverse effects of substance abuse by the mothers on their children, prenatally and post-natally. This study shows that it is also important to focus on the environment in cases where help and support are provided to presently or formerly substance abusing women raising children

## List of abbreviations

ADHD: Attention deficit and hyperactivity disorder; FAS: Fetal alcohol syndrome; FASD: Fetal alcohol spectrum disorder; NEPSY: A series of neuropsychological tests; SCWC: Special Child Welfare Clinic; TMT: Trail Making Test; WISC-III: Wechsler Intelligence Scale for Children, third version.

## Competing interests

The authors declare that they have no competing interests.

## Authors' contributions

BH conceived of the study and participated in its design, did the statistical analyses and wrote the manuscript. She has also been the doctor at the SCWC since its inception. ML and EOR participated in the design of the study, helped with statistical analyses and contributed to writing the manuscript. IS designed the tests, performed the testing and helped in analyzing the results. All authors have read and approved the final manuscript.

## Supplementary Material

Additional file 1 **Test battery used to assess neuropsychological and intellectual performance grouped by cognitive domain**. Click here for file
