# Supplementary material for: Neuropsychological screening of children of substance-abusing women attending a Special Child Welfare Clinic in Norway
Source: Subst Abuse Treat Prev Policy. 2010 Jul 20;5:17. doi: 10.1186/1747-597X-5-17 (PMC2920235; doi:10.1186/1747-597X-5-17)
Supplement: Additional file 1 — Test battery used to assess neuropsychological and intellectual performance grouped by cognitive domain. [file 1747-597X-5-17-S1.DOC]

| Neuropsychological Domain  and Constituent Items | Description |
| --- | --- |
| 1) Learning and memory | |
| Sentence repetition (NEPSY) | Repetition of sentences read aloud and of increasing length. |
| Digit span forwards (WISC-III) | Series of orally presented numbers must be repeated verbatim. |
| TMT B (Halstead–Reitan) | In TMT, B lines have to be drawn alternately between four or eight (according to  age) numbers and letters. |
| Knox Cube Test (Halstead–Reitan) | Four 1 in cubes, 4 in apart, are fastened to a thin board. The operator  moves a fifth cube touching the four cubes in a pattern that is to be copied. |
| 2) Visual scanning, planning and attention | |
| Picture completing (WISC-III) | The most important part missing in each of a set of coloured pictures has to be  identified. |
| Symbol search (WISC-III) | A self-paced task where one is given 2 min to complete as many symbol  discrimination items as possible. Then, one is asked to determine if a set of five  geometric symbols includes one of two geometric symbols. |
| Visual attention (NEPSY) | Cats: carry out a search for pictures of a target cat embedded at random in an  array of many different pictures Faces: carry out a search for pictures of two  target faces embedded at random in an array of many different faces. |
| Block design (WISC-III) | A set of blocks has to be arranged to replicate various patterns. |
| Mazes (WISC-III) | A set of paper forms on which the subject is required to trace a path through a  drawn maze of varying complexity. |
| 3) Executive functions | |
| TMT B–TMT A | Difference between TMT B and TMT A. |
| Digit Span Backwards (WISC-III) | Series of orally presented numbers must be repeated backwards. |
| Tower of London (NEPSY) | Problem solving involving forward planning to match arrangements of coloured  balls. |
|  |  |
| 4) Visuo-motor speed and dexterity | |
| Trail Making Test A (Halstead–Reitan) | In TMT A lines have to be drawn sequentially connecting four or eight encircled  numbers (according to age) distributed on a sheet of paper. |
| Visuo–motoric precision (NEPSY) | Line has to be drawn inside two consecutive narrower tracks without  overwriting the outer lines and without lifting the pencil. |
|  |  |
| Grooved pegboard (Halstead–Reitan) | The pegboard comprises 25 holes with randomly positioned slots. Pegs  with a key along one side must be rotated to match the hole before they can be  inserted. Time used is measured. Performed with one hand at a time. |
|  |  |
| 5)General intellectual ability | |
| Raven Coloured | Matrices of multiple choice tests of abstract reasoning. They become  increasingly difficult as progress is made through the set. |

Additional file 1. Test battery used to assess neuropsychological and intellectual performance grouped by cognitive

domain.
